# Impact of the COVID-19 pandemic on perinatal health and perinatal health inequalities in Europe: Jennifer Zeitlin

**DOI:** 10.1093/eurpub/ckac129.275

**Published:** 2022-10-25

**Authors:** J Zeitlin, M Philibert, TA Rihs, Ž Draušnik, M Gatt, HM Engiom, J Recape, K Szamotulska, H Barros, M Gissler

**Affiliations:** 1 Obstetrical Perinatal and Pediatric Epidemiology, Université Paris Cité, Inserm, Paris, France; 2 Federal Statistical Office, Neuchâtel, Switzerland; 3 Division of Public Health, Croatian Institute of Public Health, Zagreb, Croatia; 4 Directorate for Health Information and Research, Pieta, Malta; 5 Norwegian Institute of Public Health, Bergen, Norway; 6 Université Libre de Bruxelles, Brussels, Belgium; 7 National Research Institute of Mother and Child, Warsaw, Poland; 8 EPIUnit, University of Porto, Porto, Portugal; 9 Finnish Institute for Health and Welfare, Helsinki, Finland; 10 Karolinska Institutet, Stockholm, Sweden

## Abstract

**Background:**

The COVID-19 pandemic and lockdowns may adversely affect pregnancy outcomes due to disrupted healthcare provision and increased stress, anxiety and economic hardship. We assessed changes in perinatal outcomes in 2020 using population birth data in Europe.

**Methods:**

25 Countries in the Euro-Peristat Network implemented a federated analysis using routine national data. Countries generated anonymised aggregate data files using R scripts from individual-level data formatted to a common data model with 22 variables. We compared preterm birth, stillbirth, neonatal death and caesarean delivery rates in 2020 to 2015-2019 for 2 periods: full-year (FY) and pandemic (March-September [MS]). Data from October onward were not included in the MS period because potentially declining pandemic-related fertility may affect perinatal indicators. Country-specific relative risks (RR) for the periods, adjusted for linear trends, overall and by socio-economic (SES) group, were calculated and pooled using random effects meta-analysis.

**Results:**

Preterm birth rates decreased slightly (pooled RR: 0.97FY [95% confidence interval (CI) 0.95-0.99]; 0.98MS [0.96-1.00]) in 2020. Heterogeneity was high (I2FY=85%; I2MS=70%), with 5 countries experiencing significant declines. Neonatal mortality rates were unchanged (0.97FY [0.92-1.01]) while stillbirth rates were higher (1.05FY [1.01; 1.09]; 1.10MS [1.02; 1.19]). Caesarean rates were slightly raised (1.02FY [1.00-1.03]; 1.02MS [0.99-1.04], 5 countries had significant increases). Increases for stillbirth were more pronounced in the lowest (1.08FY [0.99-1.16]) versus highest SES group (1.05 FY [0.93-1.17]).

**Conclusions:**

In 2020, there was an unexpected decline in preterm birth in some countries, while increases in stillbirths and caesarean occurred in others. High country-level heterogeneity suggests that some government policies to mitigate the pandemic might have been more protective of pregnant women and newborns than others.

